# Health Impact Assessment of the 2020 Washington State Wildfire Smoke Episode: Excess Health Burden Attributable to Increased PM_2.5_ Exposures and Potential Exposure Reductions

**DOI:** 10.1029/2020GH000359

**Published:** 2021-05-01

**Authors:** Yisi Liu, Elena Austin, Jianbang Xiang, Tim Gould, Tim Larson, Edmund Seto

**Affiliations:** ^1^ Department of Environmental and Occupational Health Sciences University of Washington Seattle WA USA; ^2^ Department of Civil and Environmental Engineering University of Washington Seattle WA USA

**Keywords:** fine particulate matter, health impact assessment, mortality, preparedness, wildfires, wildland fires

## Abstract

Major wildfires starting in the summer of 2020 along the west coast of the United States made PM_2.5_ concentrations in this region rank among the highest in the world. Washington was impacted both by active wildfires in the state and aged wood smoke transported from fires in Oregon and California. This study aims to estimate the magnitude and disproportionate spatial impacts of increased PM_2.5_ concentrations attributable to these wildfires on population health. Daily PM_2.5_ concentrations for each county before and during the 2020 Washington wildfire episode (September 7–19) were obtained from regulatory air monitors. Utilizing previously established concentration‐response function (CRF) of PM_2.5_ (CRF of total PM_2.5_) and odds ratio (OR) of wildfire smoke days (OR of wildfire smoke days) for mortality, we estimated excess mortality attributable to the increased PM_2.5_ concentrations in Washington. On average, daily PM_2.5_ concentrations increased 97.1 μg/m^3^ during the wildfire smoke episode. With CRF of total PM_2.5_, the 13‐day exposure to wildfire smoke was estimated to lead to 92.2 (95% CI: 0.0, 178.7) more all‐cause mortality cases; with OR of wildfire smoke days, 38.4 (95% CI: 0.0, 93.3) increased all‐cause mortality cases and 15.1 (95% CI: 0.0, 27.9) increased respiratory mortality cases were attributable to the wildfire smoke episode. The potential impact of avoiding elevated PM_2.5_ exposures during wildfire events significantly reduced the mortality burden. Because wildfire smoke episodes are likely to impact the Pacific Northwest in future years, continued preparedness and mitigations to reduce exposures to wildfire smoke are necessary to avoid excess health burden.

## Key Points

- Elevated PM_2.5_ levels from a wildfire smoke episode were estimated to place a heavy mortality burden on counties in western Washington
- Counties in central and eastern Washington were estimated to have the highest per‐capita mortality during the wildfire smoke episode
- Reducing 40% of PM_2.5_ exposure for people below the federal poverty level was associated with 4.1 (95% CI: 0.0, 8.2) reduced all‐cause deaths

## 1 Introduction

A series of major wildfires impacted air quality in western regions of the United States in 2020. Fires in Northern California that started on August 19 were sparked by intense thunderstorms that ignited dry vegetation from the dry winter and sweltering summer. The northern California fires were followed by large fire complexes in Southern California, Oregon, and Washington. As of September 16, 2020, 22 large fires had been reported in California (2.3 million acres), 12 in Oregon (0.9 million acres), and 11 in Washington (0.7 million acres) (National Interagency Fire Center, 2020b). These wildfires are the worst ones on record on the West Coast (Audrey Carlsen, 2020) resulted in significant regional increases in concentrations of airborne particulates with a diameter of 2.5 μm or less (PM_2.5_). As wind shifted from a fire‐prone dry easterly flow to a northwesterly flow, smoke from the west coast fires that had traveled over the Pacific Ocean was transported to the Pacific Northwest. This resulted in some of the highest PM_2.5_ concentrations ever observed in Washington State.

Wildfire smoke consists of a mixture of air pollutants, including particulate matter, carbon monoxide, nitrogen oxides, acrolein, formaldehyde, benzene, benzo[a]pyrene, and dibenz[a,h]anthracene. Composition can vary from fire to fire, depending on the fuel (the type of vegetation, whether it burns through a town or structures), temperature, and aging in the atmosphere (Balmes, 2018). The particle size of smoke is approximately 0.4–0.7 μm in diameter. These particle sizes are harmful to human health because they are sufficiently small to be inhaled deep into the lung (Balmes, 2018).

An open question remains as to the relative toxicity of wildfire smoke as compared to other sources of ambient particles. A number of studies have estimated the acute health effects of PM_2.5_ exposures during wildfire smoke episodes (Aguilera, Corringham, Gershunov, & Benmarhnia, 2021; Aguilera, Corringham, Gershunov, Leibel, & Benmarhnia, 2021; DeFlorio‐Barker, Crooks, Reyes, & Rappold, 2019; Jones et al., 2020; Liu, Pereira, Uhl, Bravo, & Bell, 2015; Reid et al., 2016; Reid et al., 2019; Wettstein et al., 2018). Systematic reviews provide consistent evidence for the positive associations between wildfire smoke exposures and all‐cause mortality and respiratory health (Liu et al., 2015; Reid et al., 2016; Youssouf et al., 2014). Recent publications provide cumulative evidence for the association between wildfire smoke exposure and adverse cardiovascular outcomes in California (Jones et al., 2020; Wettstein et al., 2018) as well as nationally in the United States (DeFlorio‐Barker et al., 2019). Moreover, within Washington State, studies have suggested significant associations of wildfire smoke exposures with increased non‐accidental mortality (Doubleday et al., 2020) and hospital admissions for respiratory diseases (Gan et al., 2017). During the 2020 wildfire smoke episode, a report showed increased hospital emergency room visits for people with respiratory issues in the Seattle area and excess numbers of emergency medical service calls in Snohomish County (Moorer, 2020).

Given historic wildfire smoke events in Washington State, governmental agencies and research groups have recommended interventions to reduce the burden of excess exposure to air pollution. Washington State recommends people to shelter inside during wildfire smoke episodes (Washington Department of Ecology, Washington Department of Health, U.S. Forest Service, & State, 2020). At the national level, the US Environmental Protection Agency (EPA) has developed a map of fire and smoke (Interagency Wildland Fire Air Quality Response Program [IWFAQRP] and the US Environmental Protection Agency) providing real‐time PM_2.5_ data to inform public behaviors. Although existing studies have quantified deaths resulting from increased PM_2.5_ levels during wildfire smoke episodes, these have not estimated the avoidable health burden from reducing air pollution exposures by behavior changes and other interventions. The use of portable air cleaners (PAC) (Barn et al., 2016) and masks (Sbihi, Nicas, & Rideout, 2014) during wildfire smoke episode are emerging as a potentially effective public interventions. However, the relative impacts of such interventions are not well understood, in part, because the burden of increased PM_2.5_ exposure and associated health impact are not quantified for the 2020 wildfire smoke episode in Washington.

While health impact analyses have not been conducted for the most recent 2020 Washington wildfire smoke episode, previous studies have attempted to quantify the population health burden of wildfire smoke exposures in other contexts (Fann et al., 2018; Johnston et al., 2011; Matz et al., 2020; Zou et al., 2019). It was estimated that more than 0.3 million deaths were attributable to landscape fire smoke exposure each year globally (Johnston et al., 2012); 54–240 and 570–2,500 premature mortalities per year were attributable to short‐term and long‐term exposure, respectively to wildfire PM_2.5_ in Canada (Matz et al., 2020); and 1,500–2,500 premature deaths each year were attributable to short‐term exposure to wildfire PM_2.5_ in the United States from 2008 to 2012 (Fann et al., 2018). However, none of these health impact assessments had estimated the impact of reducing exposure through exposure interventions.

It is important to note the context for the 2020 wildfire smoke episode, as it is different from other wildfire seasons due to the coincidence of the COVID‐19 pandemic, which creates challenges for estimating health impacts from wildfire smoke events. We discuss these issues in detail later; however, there may be differences in exposure compared to previous wildfire smoke events due to changes in behaviors to avoid the spread of SARS‐CoV‐2, and differences in susceptibility to respiratory health effects with the co‐exposure to SARS‐CoV‐2 infection and wildfire smoke.

The goal of this study is to understand the magnitude and disproportionate spatial impacts of increased PM_2.5_ concentrations on population health attributable to the 2020 wildfire smoke episode in Washington, and to estimate the potential benefits of interventions. Previous health impact assessments employed chemistry‐transport model and satellite‐based measurements (Fann et al., 2018; Johnston et al., 2012; Zou et al., 2019) to predict PM_2.5_ concentrations during wildfire smoke episodes. These studies relied on the concentration‐response functions (CRFs) of the total PM_2.5_ instead of wildfire‐PM_2.5_, assuming that people had no behavior change during wildfire smoked episodes and that the toxicity of wildfire‐PM_2.5_ was similar to the total PM_2.5_. By utilizing empirical PM_2.5_ measurements, the CRF of total PM_2.5_ as well as the odds ratio (OR) of wildfire smoke days for mortality, we estimate increases in all‐cause, and cause‐specific mortality (respiratory mortality) for Washington, assess avoided deaths when reducing PM_2.5_ exposure by interventions, and discuss implications for public health response activities. Wildfire smoke episodes have impacted the Pacific Northwest region historically, and may continue to impact the region in future with even increased frequency and length of wildfire seasons (Westerling, 2016). Therefore, the impact estimates reported here are meant to help prepare for and inform proactive measures to reduce smoke‐induced health effects of poor air quality in future wildfires smoke episodes.

## 2 Methods

### 2.1 PM_2.5_ Exposures

We obtained the daily PM_2.5_ concentration in each of the 39 counties in Washington from Washington's Air Monitoring Network (Washington Department of Ecology) (Figure S1). Counties with regulatory air monitors were assigned the daily average PM_2.5_ concentrations from available monitors in each county. A total of six counties with small population sizes (102,245 residents in total, accounting for 1.37% of the total population in Washington) had no regulatory monitoring site. The six counties without regulatory monitors (Douglas, Lincoln, Pend Oreille, San Juan, Skamania, and Wahkiakum) were assigned the daily average PM_2.5_ concentrations of neighboring counties. The PM_2.5_ level during the wildfire smoke episode was defined as the daily PM_2.5_ concentrations from September 7 to 19, 2020. Given the seasonal variation of air pollutants, the baseline PM_2.5_ level was defined as the daily PM_2.5_ concentrations of the same period in 2019 (September 7–19, 2019)—a period with limited wildfire smoke impacts (National Interagency Fire Center, 2020a). Compared with the ambient PM_2.5_ levels during the wildfire smoke episodes, PM_2.5_ concentrations during 2019 were generally low and stable (mean: 6.0 μg/m^3^, SD: 4.6 μg/m^3^) in Washington based on continuously monitored data and a previous study (Huang et al., 2021).

### 2.2 Short‐Term Health Impact Assessment

We performed a health impact assessment for Washington to estimate the excess mortality attributable to increased PM_2.5_ during the wildfire smoke episode. The attributable fraction (AF) method was used to estimate the increased daily mortality following the equations below:

(1) $$\text{AF}=\left\{\begin{array}{l} 1-\;e^{-\beta \Delta c}\left(\text{CRF}\;\text{of}\;\text{total}\;{\text{PM}}_{2.5}\right)\\ \frac{\text{OR}-1}{\text{OR}}\;(\text{OR}\;\text{of}\;\text{wildfire}\;\text{smoke}\;\text{days}) \end{array}\right.$$

(2) $$\Delta Y\;=\;AF\;\times Y_{0}\times \text{Pop}$$

where AF is the attributable fraction of health events attributable to PM_2.5_ exposure; *β* is the cause‐specific coefficient of the CRF of PM_2.5_ for mortality from previous evidence; Δ*c* is the change in daily PM_2.5_ due to the wildfire smoke episode in each county (differences between PM_2.5_ concentrations during the wildfire smoke episode and the baseline period); OR is the odds ratio of wildfire smoke days for mortality; Δ*Y* is the estimated health impact of PM_2.5_ attributable to the wildfire smoke by county; *Y*_0_ is the baseline cause‐specific mortality rate in 2019 for each county; Pop is the exposed population by county.

Despite the growing number of studies attempting to investigate the health impacts of PM_2.5_ exposure attributable to wildfire smoke, there has been no established CRF of wildfire PM_2.5_ and mortality to date in Washington. A previous case‐crossover analysis estimated the odds of cause‐specific mortality on wildfire smoke days compared to nonwildfire smoke days based on PM_2.5_ concentrations in Washington, which showed an odds ratio of 1.02 (95% CI: 1.00, 1.05) for all‐cause mortality and 1.09 (95% CI: 1.00, 1.18) for respiratory mortality (Doubleday et al., 2020). As such, the Doubleday study took all the smoky days as the same regardless of the variations in PM_2.5_ levels. However, the observed PM_2.5_ levels in that study from 2006 to 2017 (26.4 ± 31.9 μg/m^3^) were 70% lower than the PM_2.5_ concentrations during the 2020 wildfire smoke episode. Thus, using such ORs likely underestimates the excess deaths attributable to wildfire‐related PM_2.5_ in 2020 in Washington. There are established CRFs for ambient PM_2.5_ from all sources in the western United States. A time‐series analysis estimated the associations between ambient total PM_2.5_ exposures and daily mortality and reported that a 0.50% (95% CI: 0.00, 1.01) increase in all‐cause mortality per 10 μg/m^3^ increase in PM_2.5_ in the West Coast region (i.e., Washington, Oregon, and California) (Zanobetti & Schwartz, 2009). The observed PM_2.5_ levels in that study (up to more than 100 μg/m^3^) were comparable to the PM_2.5_ levels during this wildfire smoke episode in Washington. Additionally, the approximately linear CRFs were also the health impact functions included in the US EPA health impact assessment tool (Sacks et al., 2018) that has been employed by other health impact assessments of wildfire smoke exposures in the United States (Fann et al., 2018). Utilizing this CRF may overestimate the health impacts of the wildfire‐PM_2.5_ because of public health directives encouraging people to spend more time indoors during wildfire smoke episodes. In addition, by using the CRF of total PM_2.5_ assumed similar toxicity between the total PM_2.5_ and PM_2.5_ from wildfire smoke, which may be inaccurate. We used both approaches above (CRF of total PM_2.5_ and OR of wildfire smoke days) to determine a reasonable range for the excess premature deaths attributed to increased PM_2.5_ exposure in the 2020 wildfire smoke episode in Washington.

The changes in 24‐h average PM_2.5_ during the wildfire compared to the baseline levels were calculated by county as described above. For the assessment utilizing the OR of wildfire smoke days in Washington, the wildfire smoke days were defined according to the criteria based on PM_2.5_ levels in the original study, that a daily PM_2.5_ concentration greater than 20.4 μg/m^3^, and an additional set of criteria for days between 9.0 and 20.4 μg/m^3^ (Doubleday et al., 2020). The cause‐specific annual mortality rate in 2019 for the general population in each county was obtained from the WONDER online databases (Centers for Disease Control and Prevention, 2020), which was then divided by 365 to obtain an estimated daily mortality rate. The resident population data for each county in Washington was the population estimated in 2019 from the US Census Bureau (United State Census Bureau). After performing the above calculation by county and by day during the 13‐day wildfire smoke episode, we summed the daily increased mortality to calculate the potential increased deaths in each county during the wildfire smoke episode. Finally, we assessed the total excess deaths in Washington attributable to wildfire smoke by adding up the estimated mortality in all 39 counties during the wildfire smoke episode.

### 2.3 Potential Chronic Impacts of Sustained Exposure

As climate change results in longer fire seasons (Flannigan et al., 2013), there is increasing evidence that wildfire episodes significantly contribute to the long‐term PM_2.5_ population exposures. We assessed the potential magnitude of chronic increased PM_2.5_ levels during the wildfire smoke episode on all‐cause mortality and cardiorespiratory diseases mortality following Equations 1 and 2. The increased PM_2.5_ levels during the 13‐day wildfire smoke episode was averaged over the entire year for each county in Washington. The CRFs employed in the analysis were from the extended analysis of the American Cancer Society (Krewski et al., 2009), which were also the CRFs adopted by the US EPA health impact assessment tool (Sacks et al., 2018) and had been used in previous health impact assessment of wildfire smoke exposure (Fann et al., 2018; Ford et al., 2018). That is, the all‐cause mortality and cardiorespiratory diseases mortality increased 5.60% (95% CI: 3.50, 7.80) and 12.90% (95% CI: 9.50, 16.40), respectively per 10 μg/m^3^ increase in the annual average PM_2.5_ level. Other parameters used in this analysis were the same as those used to assess short‐term health impacts.

### 2.4 Estimated Mortality Under Interventions

Different interventions to reduce wildfire smoke exposure have been recommended by health agencies in the state, including avoiding outdoor exercises, staying indoors, encouraging employers to reduce outdoor work and offer voluntary respiratory protection to workers, keeping windows closed, and using PACs (Washington Department of Ecology et al., 2020). A few studies have measured the PM_2.5_ infiltration factor (I/O_INF_) under different conditions in Washington (Allen et al., 2003; Huang et al., 2021). In particular, a natural experiment during the 2020 wildfire smoke episode in Washington found the average I/O_INF_ when sheltering indoors with windows opened was 0.92, sheltering indoors with windows closed was 0.56, using high‐efficiency particulate air (HEPA) filter portable air cleaners (PACs) was 0.19, and using do‐it‐yourself (DIY) box fan with a minimum efficiency reporting value (MERV) 13 filter was 0.3 (Xiang et al., 2021). Based on this evidence, we estimated potential reductions in excess burden that could be obtained by avoiding PM_2.5_ exposures from wildfire smoke. As a hypothetical example, we examined a future wildfire intervention policy that recognized populations living below the poverty level. Such population may be less resourced to cope with wildfire PM_2.5_, including lack of resources to purchase air cleaners. We calculated avoided all‐cause mortality during the wildfire smoke episode under plausible reduced PM_2.5_ exposures for both the total population and the targeted population below the federal poverty level in Washington (United States Census Bureau, 2019b). That is, we estimated the excess death during the wildfire smoke episode when reducing PM_2.5_ exposure by 10%, 40%, 70%, and 80%, which might be achieved by sheltering indoors, keeping windows closed, using DIY box fans with MERV 13 filters, and using HEPA filter PACs. In the case of poverty‐targeted scenario (e.g., by a state policy that improves access to interventions through incentives or subsidies), we assumed that reducing the PM_2.5_ exposure by 10%–80% only occurred for the proportion of the population in each county below the poverty line.

### 2.5 Additional Sensitivity Analyses

In addition to considering uncertainties in the exposure‐response relationship as described above using different CRF and OR‐based approaches, two other sensitivity analyses were conducted to assess variation and possible bias in the health impact estimates. One focused on exposure spatial variation, while the other addressed possible bias from nephelometer data.

First, to examine the potential for spatial exposure variation to affect results of the health impact assessment, we conducted Monte Carlo simulations to estimate the distribution of the PM_2.5_ concentrations for each county during each day of the wildfire smoke episode. The relative standard deviation (RSD) was calculated to characterize the spatial variation of daily PM_2.5_ during each day of the 13‐day wildfire smoke episode from counties with multiple monitoring sites (18 in total). The averaged RSD from the 18 counties was applied to the remaining 21 counties with a single or no monitoring site to calculate the spatial variation of PM_2.5_ during each day of the wildfire smoke episode. As the distribution of PM_2.5_ concentrations was right‐skewed, 5,000 random draws were performed from the log‐normal distribution of PM_2.5_ concentrations for each county in each day of the wildfire smoke episode. Then the daily mean and 95% confidence intervals of PM_2.5_ levels for each county in each day during the wildfire smoke episode were calculated based on the 5,000 random samples. Following the same health impact assessment procedures, increased mortality attributable to wildfire smoke in Washington was estimated.

Second, a sensitivity analysis was conducted to examine the potential bias from including nephelometer data in the analysis. Nephelometers are used at different sites in the regulatory air monitoring network in Washington. Although they are normally calibrated to US EPA federal reference method (FRM) measurements, there is the potential that these calibrations may not be appropriate for the unusually high PM_2.5_ concentrations observed during a wildfire smoke episode. The Washington Department of Ecology paired nephelometers and FRM measurements at eight monitoring sites across the state during the 2020 wildfire smoke episode in an attempt to get a statewide calibration equation for nephelometer measurements during wildfire smoke episodes. With information provided by the Washington Department of Ecology (Schulte, 2021), we conducted an additional sensitivity analysis, computing daily PM_2.5_ concentrations with the calibration equation from the 2020 wildfire season for nephelometer measurements. The 2020 wildfire calibration equation was based on PM_2.5_ measurements over 20 μg/m^3^. Comparing calibrated PM_2.5_ measurements using the 2020 wildfire calibration equation and the original calibration equation, the eight paired nephelometers consistently overestimated PM_2.5_ concentrations by 20.7% relative to the original calibration equation during the 2020 wildfire smoke episode. We computed daily PM_2.5_ concentrations for each county after adjusting the nephelometer measurements when PM_2.5_ levels were over 20 μg/m^3^. Following the same health impact assessment procedures, increased mortality attributable to wildfire smoke in Washington was estimated by considering this bias of nephelometer measurements. All the computation was conducted in R (Version 4.0.3) and RStudio^®^ (Version 1.1.456). The seed value was set to 123 in R in every random number generation to make the data analysis reproducible.

## 3 Results

### 3.1 PM_2.5_ Concentrations

**Figure 1:**
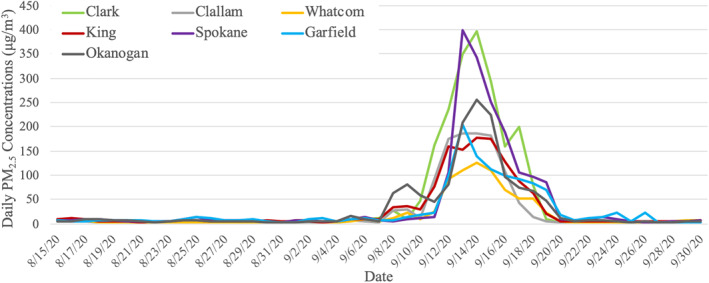
Time series plot of daily PM_2.5_ concentrations in selected counties before, during and after the 2020 wildfire smoke episode in Washington.

Figure 1 shows PM_2.5_ concentrations in selected counties before, during and after the 2020 wildfire smoke episode in Washington. PM_2.5_ levels started to climb on September 7 and returned to normal around September 19–20. The average incremental increase in PM_2.5_ concentrations during the wildfire smoke episode was 97.1 μg/m^3^ with large variations (SD = 28.5 μg/m^3^) (Table 1). Klickitat county located on the border of Washington and Oregon was heavily impacted by smoke, where PM_2.5_ concentrations showed an average of 70‐fold increase over the baseline PM_2.5_ levels (Table S1).

**Table 1:** *Summary of PM*_*2.5*_ *Concentrations in Washington Before and During the Wildfires, 2020*

	PM_2.5_ concentrations (μg/m^3^)					
	Mean	SD	Median	Range	Minimum	Maximum
Baseline (2019)	3.1	0.8	3.0	3.6	1.4	5.0
During the wildfire smoke episode	100.1	28.5	98.9	165.0	50.5	215.5
Increases	97.1	28.5	94.5	165.7	46.5	212.3

### 3.2 Short‐Term Excess Mortality Burden

**Figure 2:**
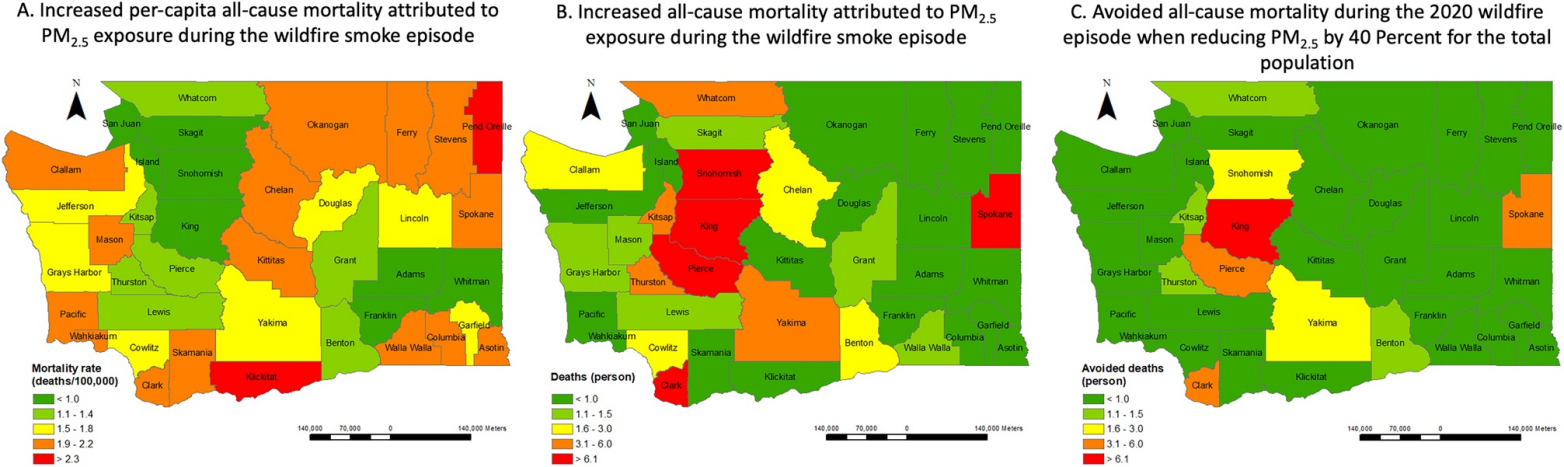
Estimated excess and avoided all‐cause mortality by county with the CRF of total PM_2.5_. Panels (a) and (b) are the estimated all‐cause mortality burden attributed to the wildfire smoke exposure for counties in Washington; (c) shows the potentially avoided all‐cause mortality burden when reducing PM_2.5_ exposure by 40% during the wildfire smoke episode. CRF, concentration‐response function.

According to our health impact assessment using the CRF for total PM_2.5_, the 13‐day exposure to wildfire smoke exposure may have led to 92.2 (95% CI: 0.0, 178.7) cases of excess all‐cause mortality. That is, 4.4% (95% CI: 0%, 8.6%) increase in all‐cause mortality associated with this wildfire smoke episode as compared to a reference periods with limited impacts from wildfire smoke. With the alternative OR of wildfire smoke days approach, we estimate 38.4 (95% CI: 0.0, 93.3) cases of all‐cause mortality and 15.71 (95% CI: 0.0, 27.9) cases of respiratory mortality attributable to the wildfire smoke episode in Washington. The mortality burden of wildfire smoke exposure was the largest in King county, due to it having the largest exposed population. Whereas, on a per‐capita basis, Klickitat county had the highest estimated burden of all‐cause mortality (increased deaths per 100,000 persons), due to the very high PM_2.5_ exposures (Table S2). In general, the wildfire smoke episode placed heavy total mortality burden on counties with large population (e.g., King County, Snohomish County, Pierce County), while counties in central and eastern Washington experienced the most severe air pollution during the wildfire smoke episode (Table S1, Figures 2a and 2b).

### 3.3 Potential Chronic Mortality Burden

As wildfire smoke episodes might significantly affect long‐term PM_2.5_ levels, we assessed the chronic impact of the increased PM_2.5_ levels during the wildfire smoke episode for each county in Washington (Table S3). This 13‐day wildfire smoke episode contributed to a mean increase of 3.5 (SD: 1.4) μg/m^3^ in the annual average of PM_2.5_, which translated to an estimated increase of 1076.0 (95% CI: 672.8, 1497.9) cases of all‐cause mortality and 930.5 (95% CI: 685.8, 1181.9) cases of cardiorespiratory mortality. That is a modeled increase of 1.8% (95% CI: 1.2, 2.6) more all‐cause deaths and 4.3% (95% CI: 3.1, 5.4) more cardiorespiratory deaths.

### 3.4 Avoidable Mortality Under Interventions

The potential value, presented as avoided mortality, of implementing interventions to reduce PM_2.5_ exposures by 10%–80% for all the residents living in Washington during the wildfire smoke episode is shown in Table 2. Figure 2c presents estimated avoided mortality when reducing PM_2.5_ exposure by 40% for the total population in Washington during the 2020 wildfire smoke episode. If intervention policies specifically targeted people living below the poverty level, 4.1 (95% CI: 0.0, 8.2) cases of all‐cause mortality can be avoided if reducing 40% of PM_2.5_ exposures for them during the wildfire smoke episode. Counties with larger populations living below the poverty level would benefit from these targeted interventions (e.g., Whatcom county). The estimated avoided all‐cause mortality when reducing PM_2.5_ exposure for the population below the poverty level in each county can be found in Table S4.

**Table 2:** *Estimated Avoided Mortality When Reducing Exposures to High PM*_*2.5*_ *Concentrations for the Total Population in Washington During the Wildfire Smoke Episode*

Reduced PM_2.5_ exposures	CRF of total PM_2.5_	OR of wildfire smoke days	
	Avoided all‐cause mortality (persons)	Avoided all‐cause mortality (persons)	Avoided respiratory mortality (persons)
10%	9.6 (0.0, 19.3)	1.7 (0.0, 5.5)	1.1 (0.0, 3.1)
40%	37.8 (0.0, 75.1)	4.0 (0.0, 11.0)	2.1 (0.0, 49)
70%	65.4 (0.0, 128.2)	11.2 (0.0, 28.5)	4.9 (0.0, 10.0)
80%	74.4 (0.0, 154.3)	13.9 (0.0, 35.1)	5.9 (0.0, 12.0)
100%	92.2 (0.0, 178.7)	38.4 (0.0, 93.3)	15.1 (0.0, 27.9)

### 3.5 Sensitivity Analyses Results

The sensitivity analysis using Monte Carlo simulation of exposure estimates and the CRF of total PM_2.5_ resulted in a similar point estimate with a wider 95% CI, that 92.2 (95% CI: 0.0, 260.9) more all‐cause mortality were estimated to be attributable to the increased PM_2.5_ concentrations during the wildfire smoke episode in Washington. After correcting nephelometer measurements, the average PM_2.5_ level during the wildfire smoke episode was 83.2 (SD: 34.3) μg/m^3^ (Table S5). The elevated PM_2.5_ concentrations resulted in lower estimates with the CRF of total PM_2.5_ (50.9, 95% CI: 0.0–98.8 more all‐cause deaths during the 2020 wildfire smoke episode in Washington); the nephelometer measurements correction did not impact the number of wildfire smoke days and the excess mortality estimates using the OR of wildfire smoke days.

## 4 Discussion

Our findings estimate that there was considerable excess mortality due to elevated PM_2.5_ concentrations during the 2020 wildfire smoke episode in Washington. We estimate 92.2 (95% CI: 0.0, 178.7) cases of increased all‐cause mortality with the CRF of total PM_2.5_, and 38.4 (95% CI: 0.0, 93.3) increased all‐cause mortality and 15.1 (95% CI: 0.0, 27.9) increased respiratory mortality with the OR of wildfire smoke days occurred during this wildfire smoke episode compared to typical periods with limited impact by wildfire smoke. The short‐term excess mortality associated with this wildfire smoke episode is large due to the relatively large increase in PM_2.5_ concentrations, not commonly observed in this part of the world. We found central and eastern Washington was heavily impacted by the wildfire smoke and was estimated to have the largest increment in per‐capita all‐cause mortality. The excess mortality burden might be avoided with potential behavior changes and interventions. With 40% of PM_2.5_ exposures reduced for the total population living in Washington, a total of 37.8 (95% CI: 0, 75.1) all‐cause mortality cases would be potentially avoided; with the same intervention, but targeting only the population living below the poverty level, 4.1 (95% CI: 0.0, 8.2) cases of all‐cause mortality might be avoided during the wildfire smoke episode. Although recent studies found positive associations between wildfire smoke exposure and increased cardiovascular symptoms (DeFlorio‐Barker et al., 2019; Jones et al., 2020; Wettstein et al., 2018), the OR we adopted from a 12‐year case‐crossover study in Washington suggested insignificant associations between wildfire smoke exposures and cardiovascular mortality (Doubleday et al., 2020). It is possible that the impact of wildfire smoke exposures on cardiovascular health is less lethal and therefore that significant associations are only observed for cardiovascular symptoms instead of mortality. Further work is necessary to refine the estimate of wildfire exposure on cardiovascular health

We used both the CRF of total PM_2.5_ and OR of wildfire smoke days approaches in the health impact assessment, and found larger estimated excess mortality attributable to increased PM_2.5_ exposure during the wildfire smoke episode when utilizing the CRF of total PM_2.5_ (Zanobetti & Schwartz, 2009) than using the OR of wildfire smoke days (Doubleday et al., 2020). Using the CRF of total PM_2.5_ assumes similar toxicity between the total PM_2.5_ and PM_2.5_ from wildfire smoke. Previous studies showed mixed results for the health effects of the general PM_2.5_ and wildfire‐PM_2.5_. Studies in the United States (DeFlorio‐Barker et al., 2019) and Finland (Hänninen et al., 2009) found similar associations of hospitalization and mortality between wildfire smoke PM_2.5_ and PM_2.5_ from other sources; however, recent studies in California found greater impacts of wildfire‐specific PM_2.5_ on respiratory health than PM_2.5_ from other sources (Aguilera, Corringham, Gershunov, & Benmarhnia, 2021; Aguilera et al., 2021). Additionally, several possible reasons may explain the lower estimated mortality burden when using the OR of wildfire smoke days than using the CRF of total PM_2.5_. First, the observed PM_2.5_ concentration in the wildfire‐PM_2.5_ study (Doubleday et al., 2020) (26.4 ± 31.9 μg/m^3^) were lower than the observed PM_2.5_ levels in the study of total PM_2.5_ (Zanobetti & Schwartz, 2009) and than the PM_2.5_ levels during the 2020 wildfire smoke episode (100.1 ± 28.5 μg/m^3^). Therefore, by not accounting for dose, the OR of wildfire smoke days approach may underestimate the excess mortality attributable to wildfire‐PM_2.5_. Second, the OR of wildfire smoke days was from a study estimating ORs of mortality on wildfire smoke days compared to nonwildfire smoke days, rather than the associations between total PM_2.5_ concentrations and daily mortality. Therefore, the OR of wildfire smoke days may already account for some behavior changes (e.g., avoiding outdoor exercises, sheltering indoors) during wildfire seasons.

State and regional air quality stakeholders have taken steps in recent years to improve preparedness and to encourage exposure reductions for wildfire smoke events. The state governor's office released a series of proclamations (Washington Governor, 2020) aiming at addressing the impacts of this particular wildfire episode. Additionally, several agencies (Department of Ecology, Department of Health, US Forest Service, regional Air Quality Agencies, County Health Departments, and Tribes) collaborated in previous years to provide information to the public via a website (Washington Department of Ecology et al., 2020). Behavior changes and interventions have been proposed and recommended by different agencies to support public health. People, especially children, pregnant women, the elderly and people with preexisting diseases, are recommended to stay indoors. The public is also advised to limit the infiltration of smoke into indoor environment, reduce indoor pollution sources, and in some cases, use personal protective equipment when they have to be outdoors during wildfire smoke episodes. HEPA and heating, ventilation, and air conditioning (HVAC) filters are available at local stores to help reduce indoor particulate matter levels; commercially available PAC can also effectively reduce particle levels even in indoor spaces without central HVAC systems (Fisk & Chan, 2017). A recent natural experiment during the 2020 wildfire smoke episode in Seattle found that using a HEPA PAC and DIY box fan with a MERV 13 filter significantly reduced the PM_2.5_ infiltration factor indoors to 0.19 and 0.3, respectively (Xiang et al., 2021). We estimated that reducing PM_2.5_ exposure by 40% with interventions for all the residents in Washington had the potential to reduce 37.8 (95% CI: 0, 75.1) cases of all‐cause deaths (avoided all‐cause mortality was 3.6, 95% CI: 0.0–9.0 and respiratory mortality was 1.5, 95% CI: 0.0–2.9 with the OR of wildfire smoke days). Because wildfire smoke is likely to continue impacting the Pacific Northwest in future years, effective strategies should be implemented to reduce exposures and to avoid the excess health burden from wildfire smoke. This is especially the case for counties that tend to be more impacted during wildfire smoke episodes, such as those in central and eastern Washington. These regions were also identified as the most impacted by wildfire smoke exposures during the 2017 wildfire season in Washington (Zou et al., 2019). Therefore, strategies and interventions should be prioritized for central and eastern Washington as a matter of environmental justice.

While counties in central and eastern Washington were exposed to the highest level of PM_2.5_ during this wildfire smoke episode, these counties also have a large number of underserved communities and outdoor workers (e.g., agricultural population), who may need additional attention (Table S4 presents estimated population living below the poverty level and their proportion to the total population). Underserved families may not be able to afford air cleaners or filtration systems at home, may have higher rates of preexisting health conditions that make them more vulnerable to smoke exposure, and may lack access to health care services to cope with the health impacts of poor air quality. For instance, future interventions may be prioritized for people living below the poverty level such that each household will be given a HEPA‐filter PAC. With the average household size of 2.55 in Washington (United States Census Bureau, 2019a), the total estimated population living below the poverty of 785,244, and the price for each HEPA‐PAC of $150, the cost for purchasing HEPA‐PACs for the 307,939 households will be 46.2 million US dollars. Such intervention would have potentially resulted in a total of 4.1 (95% CI: 0.0, 8.2) cases of all‐cause mortality avoided if it reduced 40% of PM_2.5_ exposures for people living below the poverty level during the wildfire smoke episode.

Outdoor workers are exposed to not only high PM_2.5_ concentrations but also high heat during wildfire smoke episodes. While the study found the highest PM_2.5_ levels in central and eastern Washington during the wildfire smoke episode, these regions also have a large proportion of agricultural works (e.g., Klickitat, Okanogan) (Employment Security Department, 2019). A recent study found that the largest agricultural populations in Washington tended to be located in counties with the greatest high heat and PM_2.5_ exposures, and these exposures tended to coincide with months with the highest numbers of agricultural workers (harvest season during July–September). However, Washington has no occupational exposure rules specific to PM_2.5_ during wildfire smoke events (Austin, Kasner, Seto, & Spector, 2020). The particular wildfire smoke event discussed here shows the importance of enhancing capacity and access to clean indoor air spaces and personal protection during wildfire events. Findings of high wildfire smoke levels in central and eastern Washington in this study emphasizes the need for air quality interventions in these areas, which are large agricultural communities. Continued strategies and actions are needed to help the public (especially outdoor workers and underserved communities) be better prepared for future wildfire events, and to develop community resilience plans to reduce the disproportionate impacts of wildfire smoke.

The 2020 wildfire smoke episode may be difficult to analyze using hospital record data due to the coincidence with the COVID‐19 pandemic. Although there is still insufficient evidence to support this, exposure to elevated wildfire smoke may exacerbate the effects of SARS‐CoV‐2 infection (Henderson, 2020). There is clear evidence of increased acute lower respiratory infections with PM_2.5_ exposure, especially for children (Croft et al., 2018; Dominici et al., 2006; Horne et al., 2018). Evidence is also available for the delayed effect of higher PM_2.5_ levels during the wildfire season on increased influenza in the following winter flu season (Landguth et al., 2020). On the other hand, given the stay‐at‐home order to prevent the spread of SARS‐CoV‐2 during this wildfire smoke episode, fewer people may be outdoors and exposed to ambient PM_2.5_ than if there was no pandemic. For instance, many children are not currently attending school in person, which may result in less exposure to outdoor PM. However, essential workers, including many outdoor worker categories (e.g., firefighters, emergency personnel, agriculture and construction workers, delivery persons, etc.), may have been more exposed to air pollution during this period, and thus might be at higher risk for health effects.

A potential limitation of this work is that behavior changes of residents due to the COVID‐19 pandemic during the wildfire smoke episode were not accounted for. However, this study aims to understand the magnitude and disproportionate spatial impact of PM_2.5_ exposure on mortality during the wildfire smoke episode, instead of estimating specific expected responses. Another limitation is that we employed a county level exposure metric in this analysis rather than a spatially refined model. Although this may result in spatial misalignment of exposure, one advantage was that these estimates relied on in situ monitoring data, providing accurate continuous information about PM_2.5_ concentrations. To estimate the impact of this, we evaluated the size of the smoke plume from long‐range transport (Figure S2) and determined that county‐level estimates are an appropriate scale for this wildfire analysis. We conducted Monte Carlo simulations in the sensitivity analysis to estimate potential impact of smaller scale spatial variations of PM_2.5_, especially in counties with no or only one monitoring site. We also estimated the magnitude of exposure bias from nephelometer measurements in the sensitivity analysis. Because the size distribution may be different for wildfire‐PM_2.5_ and the general PM_2.5_, there is concern that nephelometers tend to overestimate PM_2.5_ concentrations by 20.7% during wildfire smoke episodes. This was corroborated by information provided by the Washington Department of Ecology. We have not yet assessed changes in state vital records, which we would anticipate to confirm the impact assessment estimates. However, the COVID‐19 pandemic and behavior changes may attenuate the hospital records analysis. We have also not yet assessed morbidity impacts. Future work should include morbidity, which may affect a greater portion of the population.

## Conflict of Interest

The authors declare they have no actual or potential competing financial interests.

## Supporting information

Supporting Information S1

## Data Availability

Data sets for this research are freely and publicly available in references throughout the Methods section, including PM2.5 data from Washington Department of Ecology (https://enviwa.ecology.wa.gov/Report/Hr24PM25SummaryNewl.), population data from United State Census Bureau (https://www.census.gov/data/datasets/time-series/demo/popest/2010s-counties-total.html) and mortality rate data from Centers for Diseases Control and Prevention (http://wonder.cdc.gov/mcd-icd10-expanded.html).
