# Supplementary material for: Health Impact Assessment of the 2020 Washington State Wildfire Smoke Episode: Excess Health Burden Attributable to Increased PM2.5 Exposures and Potential Exposure Reductions
Source: Geohealth. 2021 May 1;5(5):e2020GH000359. doi: 10.1029/2020GH000359 (PMC8101535; doi:10.1029/2020GH000359)
Supplement: Supplementary file 1 — Supporting Information S1 [file GH2-5-e2020GH000359-s001.docx]

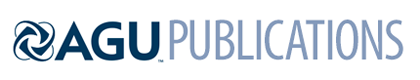


*GeoHealth*

Supporting Information for

**Health Impact Assessment of the 2020 Washington State Wildfire Smoke Episode: Excess Health Burden Attributable to Increased PM_2.5_ Exposures and Potential Exposure Reductions**

Yisi Liu^1^†, Elena Austin^1^, Jianbang Xiang^1^, Tim Gould^2^, Tim Larson^1,2^, Edmund Seto^1^

^1^. Department of Environmental and Occupational Health Sciences, University of Washington, Seattle, WA 98195, United States

^2^. Department of Civil and Environmental Engineering, University of Washington, Seattle, WA 98195, United States

**Contents of this file**

Tables S1 to S5

Figures S1 to S2

**Introduction**

This supporting information provides results of the sensitivity analysis, the baseline mortality rate, PM_2.5_ concentrations, and estimated health burden for each county, the map of the air monitoring network in Washington, and a modeled PM_2.5_ concentration in a typical day during the wildfire smoke episode.

**Table S1.** The average PM_2.5_ concentration changes during the 2020 wildfire episode and baseline mortality rate for each county.

| County | Population  (persons) | PM_2.5_ baseline  (𝜇g/m^3^) | PM_2.5_ during wildfire  (𝜇g/m^3^) | PM_2.5_ increases  (𝜇g/m^3^) | All-cause mortality rate  (deaths/100,000) | Cardiovascular mortality rate (deaths/100,000) | Respiratory mortality rate  (deaths/100,000) |
| --- | --- | --- | --- | --- | --- | --- | --- |
| Adams | 19,983 | 1.94 | 80.35 | 78.41 | 640.54 | 170.14 | NA* |
| Asotin | 22,582 | 4.75 | 87.61 | 82.86 | 1222.21 | 402.98 | 123.99 |
| Benton | 204,390 | 3.46 | 97.30 | 93.84 | 733.89 | 198.15 | 80.24 |
| Chelan | 77,200 | 4.09 | 111.15 | 107.07 | 988.34 | 277.20 | 97.15 |
| Clallam | 77,331 | 2.15 | 102.17 | 100.02 | 1475.48 | 429.32 | 147.42 |
| Clark | 488,241 | 2.16 | 116.85 | 114.69 | 793.26 | 229.60 | 74.55 |
| Columbia | 3,985 | 2.66 | 115.59 | 112.93 | 1505.65 | 401.51 | NA |
| Cowlitz | 110,593 | 2.96 | 85.61 | 82.65 | 1123.94 | 311.95 | 131.11 |
| Douglas | 43,429 | 3.48 | 106.90 | 103.42 | 766.77 | 195.72 | 69.08 |
| Ferry | 7,627 | 2.88 | 119.28 | 116.40 | 1101.35 | 183.56 | 170.45 |
| Franklin | 95,222 | 1.89 | 101.82 | 99.93 | 457.88 | 131.27 | 37.81 |
| Garfield | 2,225 | 2.61 | 80.58 | 77.97 | 1168.54 | NA | NA |
| Grant | 97,733 | 2.16 | 82.94 | 80.78 | 721.35 | 200.55 | 99.25 |
| Grays Harbor | 75,061 | 3.19 | 83.95 | 80.76 | 1212.35 | 321.07 | 138.55 |
| Island | 85,141 | 1.35 | 53.36 | 52.00 | 949.01 | 267.79 | 86.91 |
| Jefferson | 32,221 | 3.99 | 50.51 | 46.53 | 1272.46 | 350.70 | 139.66 |
| King | 2,252,782 | 4.40 | 79.48 | 75.08 | 601.88 | 173.07 | 47.10 |
| Kitsap | 271,473 | 3.28 | 84.64 | 81.36 | 871.91 | 249.75 | 75.51 |
| Kittitas | 47,935 | 2.70 | 124.96 | 122.27 | 740.59 | 219.05 | 66.76 |
| Klickitat | 22,425 | 3.21 | 215.48 | 212.26 | 918.62 | 249.72 | 98.10 |
| Lewis | 80,707 | 2.77 | 167.11 | 164.34 | 1152.32 | 349.41 | 130.10 |
| Lincoln | 10,939 | 2.97 | 86.47 | 83.50 | 1042.14 | 310.81 | 127.98 |
| Mason | 66,768 | 3.42 | 109.21 | 105.79 | 1070.87 | 304.04 | 104.84 |
| Okanogan | 42,243 | 4.99 | 110.88 | 105.89 | 1131.55 | 298.27 | 104.16 |
| Pacific | 22,471 | 2.52 | 91.54 | 89.02 | 1468.56 | 556.27 | 146.86 |
| Pend Oreille | 13,724 | 3.05 | 102.40 | 99.36 | 1136.69 | 262.31 | 167.59 |
| Pierce | 904,980 | 3.51 | 106.77 | 103.26 | 763.66 | 215.81 | 74.48 |
| San Juan | 17,582 | 2.74 | 77.60 | 74.86 | 836.08 | 187.69 | 68.25 |
| Skagit | 129,205 | 2.48 | 58.83 | 56.35 | 965.13 | 276.31 | 83.59 |
| Skamania | 12,083 | 3.10 | 96.67 | 93.57 | 877.27 | 231.73 | 99.31 |
| Snohomish | 822,083 | 2.63 | 105.62 | 102.99 | 682.53 | 174.56 | 62.04 |
| Spokane | 522,798 | 3.77 | 96.53 | 92.77 | 896.52 | 228.39 | 99.85 |
| Stevens | 45,723 | 2.33 | 125.76 | 123.43 | 999.50 | 310.57 | 111.54 |
| Thurston | 290,536 | 3.27 | 104.08 | 100.82 | 837.07 | 215.12 | 88.80 |
| Wahkiakum | 4,488 | 2.75 | 81.34 | 78.58 | 1426.02 | 401.07 | NA* |
| Walla Walla | 60,760 | 2.75 | 105.17 | 102.41 | 934.83 | 266.62 | 74.06 |
| Whatcom | 229,247 | 3.71 | 116.66 | 112.95 | 809.17 | 235.12 | 72.85 |
| Whitman | 50,104 | 2.73 | 82.32 | 79.60 | 532.89 | 171.64 | 61.87 |
| Yakima | 250,873 | 4.39 | 98.93 | 94.55 | 799.21 | 265.47 | 71.75 |

* The mortality rate was suppressed to protect privacy.

**Table S2.** The estimated short-term cause-specific mortality and mortality per 100,000 persons attributed to increased PM_2.5_ levels during the wildfire episode for each county.

| County | CRF of total PM_2.5_ | | OR of wildfire smoke days | | | |
| --- | --- | --- | --- | --- | --- | --- |
|  | All-cause mortality  (persons) | All-cause mortality rate  (deaths/100,000) | All-cause mortality  (persons) | All-cause mortality rate  (deaths/100,000) | Respiratory mortality (persons) | Respiratory mortality rate  (deaths/100,000) |
| Adams | 0.17 (0.00, 0.34) | 0.87 (0.00, 1.69) | 0.06 (0.00, 0.15) | NA* | 0.31 (0.00, 0.77) | NA* |
| Asotin | 0.43 (0.00, 0.83) | 1.89 (0.00, 3.69) | 0.19 (0.00, 0.48) | 0.09 (0.00, 0.16) | 0.86 (0.00, 2.12) | 0.38 (0.00, 0.73) |
| Benton | 2.48 (0.00, 4.82) | 1.21 (0.00, 2.36) | 1.06 (0.00, 2.61) | 0.50 (0.00, 0.96) | 0.52 (0.00, 1.27) | 0.25 (0.00, 0.47) |
| Chelan | 1.53 (0.00, 2.96) | 1.99 (0.00, 3.83) | 0.54 (0.00, 1.33) | 0.23 (0.00, 0.44) | 0.70 (0.00, 1.72) | 0.30 (0.00, 0.57) |
| Clallam | 1.56 (0.00, 3.04) | 2.02 (0.00, 3.93) | 0.68 (0.00, 1.68) | 0.30 (0.00, 0.57) | 0.88 (0.00, 2.17) | 0.38 (0.00, 0.73) |
| Clark | 9.68 (0.00, 18.36) | 1.98 (0.00, 3.76) | 2.73 (0.00, 6.73) | 1.12 (0.00, 2.14) | 0.56 (0.00, 1.38) | 0.23 (0.00, 0.44) |
| Columbia | 0.08 (0.00, 0.16) | 1.99 (0.00, 3.90) | 0.04 (0.00, 0.09) | NA* | 0.90 (0.00, 2.21) | NA* |
| Cowlitz | 1.91 (0.00, 3.73) | 1.72 (0.00, 3.37) | 0.74 (0.00, 1.83) | 0.38 (0.00, 0.72) | 0.67 (0.00, 1.65) | 0.34 (0.00, 0.65) |
| Douglas | 0.67 (0.00, 1.29) | 1.54 (0.00, 2.96) | 0.23 (0.00, 0.58) | 0.09 (0.00, 0.18) | 0.54 (0.00, 1.33) | 0.21 (0.00, 0.41) |
| Ferry | 0.16 (0.00, 0.32) | 2.15 (0.00, 4.17) | 0.06 (0.00, 0.15) | 0.04 (0.00, 0.08) | 0.78 (0.00, 1.91) | 0.52 (0.00, 1.00) |
| Franklin | 0.64 (0.00, 1.24) | 0.67 (0.00, 1.31) | 0.31 (0.00, 0.76) | 0.11 (0.00, 0.21) | 0.32 (0.00, 0.80) | 0.12 (0.00, 0.22) |
| Garfield | 0.03 (0.00, 0.06) | 1.45 (0.00, 2.84) | 0.02 (0.00, 0.04) | NA* | 0.70 (0.00, 1.72) | NA* |
| Grant | 1.08 (0.00, 2.12) | 1.11 (0.00, 2.17) | 0.50 (0.00, 1.22) | 0.30 (0.00, 0.57) | 0.51 (0.00, 1.25) | 0.30 (0.00, 0.58) |
| Grays Harbor | 1.15 (0.00, 2.25) | 1.53 (0.00, 2.99) | 0.54 (0.00, 1.34) | 0.27 (0.00, 0.52) | 0.72 (0.00, 1.78) | 0.36 (0.00, 0.69) |
| Island | 0.41 (0.00, 0.83) | 0.49 (0.00, 0.97) | 0.48 (0.00, 1.19) | 0.19 (0.00, 0.37) | 0.57 (0.00, 1.39) | 0.23 (0.00, 0.43) |
| Jefferson | 0.47 (0.00, 0.93) | 1.47 (0.00, 2.88) | 0.27 (0.00, 0.66) | 0.13 (0.00, 0.24) | 0.83 (0.00, 2.04) | 0.40 (0.00, 0.76) |
| King | 19.64 (0.00, 38.43) | 0.87 (0.00, 1.71) | 9.56 (0.00, 23.55) | 3.25 (0.00, 6.22) | 0.42 (0.00, 1.05) | 0.14 (0.00, 0.28) |
| Kitsap | 3.18 (0.00, 6.22) | 1.17 (0.00, 2.29) | 1.54 (0.00, 3.80) | 0.58 (0.00, 1.11) | 0.57 (0.00, 1.40) | 0.21 (0.00, 0.41) |
| Kittitas | 0.97 (0.00, 1.82) | 2.03 (0.00, 3.80) | 0.25 (0.00, 0.62) | 0.10 (0.00, 0.19) | 0.52 (0.00, 1.29) | 0.20 (0.00, 0.39) |
| Klickitat | 0.83 (0.00, 1.49) | 3.72 (0.00, 6.65) | 0.15 (0.00, 0.36) | 0.07 (0.00, 0.13) | 0.65 (0.00, 1.60) | 0.30 (0.00, 0.58) |
| Lewis | 1.12 (0.00, 2.20) | 1.39 (0.00, 2.73) | 0.61 (0.00, 1.49) | 0.30 (0.00, 0.57) | 0.75 (0.00, 1.85) | 0.37 (0.00, 0.70) |
| Lincoln | 0.19 (0.00, 0.37) | 1.74 (0.00, 3.38) | 0.07 (0.00, 0.18) | 0.04 (0.00, 0.08) | 0.68 (0.00, 1.67) | 0.36 (0.00, 0.69) |
| Mason | 1.39 (0.00, 2.67) | 2.08 (0.00, 4.00) | 0.47 (0.00, 1.15) | 0.20 (0.00, 0.38) | 0.70 (0.00, 1.72) | 0.30 (0.00, 0.57) |
| Okanogan | 0.81 (0.00, 1.58) | 1.92 (0.00, 3.74) | 0.31 (0.00, 0.77) | 0.12 (0.00, 0.24) | 0.74 (0.00, 1.81) | 0.29 (0.00, 0.56) |
| Pacific | 0.44 (0.00, 0.85) | 1.94 (0.00, 3.80) | 0.21 (0.00, 0.53) | 0.09 (0.00, 0.18) | 0.96 (0.00, 2.35) | 0.42 (0.00, 0.80) |
| Pend Oreille | 0.32 (0.00, 0.61) | 2.33 (0.00, 4.44) | 0.09 (0.00, 0.23) | 0.06 (0.00, 0.11) | 0.68 (0.00, 1.67) | 0.43 (0.00, 0.83) |
| Pierce | 10.05 (0.00, 19.67) | 1.11 (0.00, 2.17) | 4.87 (0.00, 12.00) | 2.07 (0.00, 3.95) | 0.54 (0.00, 1.33) | 0.23 (0.00, 0.44) |
| San Juan | 0.16 (0.00, 0.32) | 0.94 (0.00, 1.84) | 0.10 (0.00, 0.24) | 0.03 (0.00, 0.06) | 0.54 (0, .00 1.34) | 0.19 (0.00, 0.37) |
| Skagit | 1.04 (0.00, 2.07) | 0.81 (0.00, 1.60) | 0.81 (0.00, 2.00) | 0.31 (0.00, 0.58) | 0.63 (0, .00 1.55) | 0.24 (0.00, 0.45) |
| Skamania | 0.25 (0.00, 0.47) | 2.06 (0.00, 3.93) | 0.07 (0.00, 0.18) | 0.04 (0.00, 0.07) | 0.62 (0.00, 1.52) | 0.30 (0.00, 0.58) |
| Snohomish | 6.38 (0.00, 12.58) | 0.78 (0.00, 1.53) | 3.65 (0.00, 9.00) | 1.44 (0.00, 2.76) | 0.44 (0.00, 1.09) | 0.18 (0.00, 0.34) |
| Spokane | 9.50 (0.00, 18.04) | 1.82 (0.00, 3.45) | 2.54 (0.00, 6.26) | 1.23 (0.00, 2.36) | 0.49 (0.00, 1.20) | 0.24 (0.00, 0.45) |
| Stevens | 0.95 (0.00, 1.81) | 2.08 (0.00, 3.95) | 0.27 (0.00, 0.67) | 0.13 (0.00, 0.25) | 0.60 (0.00, 1.47) | 0.29 (0.00, 0.55) |
| Thurston | 3.29 (0.00, 6.45) | 1.13 (0.00, 2.22) | 1.58 (0.00, 3.90) | 0.73 (0.00, 1.40) | 0.54 (0.00, 1.34) | 0.25 (0.00, 0.48) |
| Wahkiakum | 0.09 (0.00, 0.17) | 1.93 (0.00, 3.79) | 0.04 (0.00, 0.10) | NA* | 0.93 (0.00, 2.29) | NA* |
| Walla Walla | 1.21 (0.00, 2.32) | 2.00 (0.00, 3.82) | 0.37 (0.00, 0.91) | 0.13 (0.00, 0.24) | 0.61 (0.00, 1.50) | 0.21 (0.00, 0.40) |
| Whatcom | 3.17 (0.00, 6.13) | 1.38 (0.00, 2.67) | 1.21 (0.00, 2.97) | 0.47 (0.00, 0.90) | 0.53 (0.00, 1.30) | 0.21 (0.00, 0.39) |
| Whitman | 0.27 (0.00, 0.53) | 0.54 (0.00, 1.06) | 0.13 (0.00, 0.32) | 0.07 (0.00, 0.13) | 0.26 (0.00, 0.64) | 0.13 (0.00, 0.25) |
| Yakima | 4.49 (0.00, 8.59) | 1.79 (0.00, 3.43) | 1.41 (0.00, 3.48) | 0.55 (0.00, 1.06) | 0.56 (0.00, 1.39) | 0.22 (0.00, 0.42) |

* Increased deaths were not estimated due to data unavailability.

**Table S3.** The estimated long-term cause-specific mortality and mortality per 100,000 persons attributed to increased PM2.5 levels during the wildfire episode for each county.

| County | Annual average PM_2.5_ increased  (𝜇g/m^3^) | All-cause mortality  (persons) | Cardiorespiratory mortality  (persons) | All-cause mortality rate  (deaths/100,000) | Cardiorespiratory mortality rate  (deaths/100,000) |
| --- | --- | --- | --- | --- | --- |
| Adams | 2.79 | 2.00 (1.25, 2.78) | 1.22 (0.90, 1.55) | 10.01 (6.26, 13.93) | 6.12 (4.51, 7.77) |
| Asotin | 3.21 | 4.96 (3.10, 6.90) | 4.91 (3.62, 6.24) | 21.94 (13.72, 30.55) | 21.76 (16.04, 27.65) |
| Benton | 3.43 | 28.77 (17.99, 40.05) | 25.10 (18.5, 31.89) | 14.08 (8.80, 19.60) | 12.28 (9.05, 15.60) |
| Chelan | 4.22 | 18.00 (11.26, 25.06) | 15.68 (11.56, 19.91) | 23.32 (14.58, 32.46) | 20.31 (14.97, 25.79) |
| Clallam | 2.84 | 18.10 (11.32, 25.2) | 16.27 (11.99, 20.67) | 23.40 (14.63, 32.58) | 21.04 (15.51, 26.73) |
| Clark | 5.33 | 115.47 (72.23, 160.71) | 101.72 (75.00, 129.16) | 23.65 (14.79, 32.92) | 20.83 (15.36, 26.45) |
| Columbia | 2.73 | 0.92 (0.57, 1.27) | 0.56 (0.41, 0.71) | 22.97 (14.36, 31.99) | 14.09 (10.39, 17.91) |
| Cowlitz | 3.17 | 22.05 (13.79, 30.70) | 20.00 (14.74, 25.41) | 19.94 (12.47, 27.76) | 18.08 (13.32, 22.97) |
| Douglas | 4.21 | 7.85 (4.91, 10.92) | 6.23 (4.59, 7.91) | 18.07 (11.3,0 25.15) | 14.34 (10.57, 18.22) |
| Ferry | 4.06 | 1.91 (1.19, 2.65) | 1.41 (1.04, 1.79) | 24.99 (15.62, 34.79) | 18.47 (13.61, 23.46) |
| Franklin | 3.03 | 7.38 (4.62, 10.28) | 6.27 (4.62, 7.97) | 7.75 (4.85, 10.79) | 6.59 (4.85, 8.37) |
| Garfield | 2.55 | 0.37 (0.23, 0.52) | NA* | 16.69 (10.44, 23.24) | NA* |
| Grant | 3.19 | 12.56 (7.85, 17.49) | 12.01 (8.85, 15.26) | 12.85 (8.04, 17.90) | 12.29 (9.06, 15.61) |
| Grays Harbor | 2.60 | 13.26 (8.29, 18.46) | 11.56 (8.52, 14.69) | 17.66 (11.04, 24.59) | 15.4 0(11.35, 19.57) |
| Island | 1.03 | 4.68 (2.93, 6.52) | 4.03 (2.97, 5.12) | 5.50 (3.44, 7.66) | 4.73 (3.49, 6.01) |
| Jefferson | 2.37 | 5.44 (3.4,0 7.58) | 4.83 (3.56, 6.13) | 16.89 (10.56, 23.52) | 14.98 (11.04, 19.03) |
| King | 2.99 | 226.80 (141.80, 315.77) | 190.87 (140.65, 242.51) | 10.07 (6.29, 14.02) | 8.47 (6.24, 10.76) |
| Kitsap | 2.77 | 36.63 (22.90, 51.00) | 31.44 (23.16, 39.94) | 13.49 (8.44, 18.79) | 11.58 (8.53, 14.71) |
| Kittitas | 5.92 | 11.75 (7.35, 16.34) | 10.41 (7.68, 13.21) | 24.50 (15.33, 34.1) | 21.71 (16.01, 27.56) |
| Klickitat | 9.22 | 10.59 (6.63, 14.72) | 9.19 (6.78, 11.65) | 47.21 (29.55, 65.66) | 40.96 (30.24, 51.94) |
| Lewis | 2.47 | 12.88 (8.05, 17.93) | 12.33 (9.09, 15.67) | 15.96 (9.98, 22.22) | 15.28 (11.26, 19.42) |
| Lincoln | 3.48 | 2.22 (1.39, 3.09) | 2.15 (1.58, 2.73) | 20.29 (12.69, 28.24) | 19.65 (14.48, 24.96) |
| Mason | 4.07 | 16.28 (10.18, 22.66) | 14.29 (10.53, 18.15) | 24.38 (15.24, 33.94) | 21.40 (15.77, 27.18) |
| Okanogan | 3.53 | 9.43 (5.90, 13.13) | 7.72 (5.69, 9.80) | 22.33 (13.96, 31.09) | 18.26 (13.46, 23.20) |
| Pacific | 2.73 | 5.03 (3.15, 7.01) | 5.54 (4.08, 7.04) | 22.39 (14.00, 31.18) | 24.67 (18.18, 31.34) |
| Pend Oreille | 4.37 | 3.81 (2.38, 5.30) | 3.31 (2.44, 4.20) | 27.77 (17.37, 38.65) | 24.13 (17.79, 30.64) |
| Pierce | 3.00 | 116.04 (72.55, 161.57) | 101.48 (74.78, 128.94) | 12.82 (8.02, 17.85) | 11.21 (8.26, 14.25) |
| San Juan | 2.30 | 1.89 (1.18, 2.64) | 1.33 (0.98, 1.70) | 10.78 (6.74, 15.01) | 7.59 (5.59, 9.65) |
| Skagit | 1.70 | 11.87 (7.42, 16.53) | 10.19 (7.51, 12.95) | 9.19 (5.74, 12.8) | 7.89 (5.81, 10.02) |
| Skamania | 4.99 | 2.95 (1.85, 4.11) | 2.56 (1.89, 3.25) | 24.45 (15.29, 34.03) | 21.20 (15.63, 26.92) |
| Snohomish | 2.33 | 73.26 (45.80, 102.01) | 58.44 (43.06, 74.26) | 8.91 (5.57, 12.41) | 7.11 (5.24, 9.03) |
| Spokane | 4.32 | 113.04 (70.70, 157.32) | 95.09 (70.11, 120.74) | 21.62 (13.52, 30.09) | 18.19 (13.41, 23.10) |
| Stevens | 4.43 | 11.31 (7.07, 15.73) | 10.97 (8.09, 13.93) | 24.73 (15.46, 34.41) | 23.99 (17.69, 30.47) |
| Thurston | 2.79 | 37.95 (23.72, 52.83) | 31.70 (23.36, 40.27) | 13.06 (8.17, 18.18) | 10.91 (8.04, 13.86) |
| Wahkiakum | 2.79 | 1.00 (0.62, 1.39) | 0.65 (0.48, 0.82) | 22.26 (13.92, 31.00) | 14.41 (10.62, 18.31) |
| Walla Walla | 4.50 | 14.3 (8.95, 19.91) | 11.98 (8.83, 15.22) | 23.54 (14.72, 32.77) | 19.72 (14.54, 25.05) |
| Whatcom | 3.57 | 37.09 (23.19, 51.63) | 32.45 (23.92, 41.22) | 16.18 (10.12, 22.52) | 14.16 (10.43, 17.98) |
| Whitman | 2.06 | 3.08 (1.92, 4.29) | 3.10 (2.29, 3.94) | 6.14 (3.84, 8.55) | 6.19 (4.56, 7.87) |
| Yakima | 4.73 | 53.06 (33.19, 73.86) | 51.46 (37.94, 65.35) | 21.15 (13.23, 29.44) | 20.51 (15.12, 26.05) |

* Increased deaths were not estimated due to data unavailability.

**Table S4.** The estimated avoided all-cause mortality when reducing PM_2.5_ levels for the population below poverty level during the wildfire episode for each county.

| County | Population below poverty level (persons) | percent of people below poverty level (%) | Avoided all-cause mortality when reducing 10% of PM_2.5_ exposures (persons) | Avoided all-cause mortality when reducing 40% of PM_2.5_ exposures (persons) | Avoided all-cause mortality when reducing 70% of PM_2.5_ exposures (persons) | Avoided all-cause mortality when reducing 80% of PM_2.5_ exposures (persons) |
| --- | --- | --- | --- | --- | --- | --- |
| Adams | 4,951 | 25.6 | 0.00 (0.00, 0.01) | 0.02 (0.00, 0.03) | 0.03 (0.00, 0.06) | 0.03 (0.00, 0.07) |
| Asotin | 2,795 | 12.6 | 0.01 (0.00, 0.01) | 0.02 (0.00, 0.04) | 0.04 (0.00, 0.07) | 0.04 (0.00, 0.08) |
| Benton | 23,336 | 11.9 | 0.03 (0.00, 0.06) | 0.12 (0.00, 0.23) | 0.20 (0.00, 0.39) | 0.23 (0.00, 0.45) |
| Chelan | 8,811 | 11.7 | 0.02 (0.00, 0.04) | 0.07 (0.00, 0.14) | 0.12 (0.00, 0.24) | 0.14 (0.00, 0.27) |
| Clallam | 10,768 | 14.5 | 0.02 (0.00, 0.05) | 0.09 (0.00, 0.18) | 0.15 (0.00, 0.30) | 0.18 (0.00, 0.34) |
| Clark | 43,384 | 9.3 | 0.09 (0.00, 0.18) | 0.36 (0.00, 0.70) | 0.61 (0.00, 1.19) | 0.70 (0.00, 1.34) |
| Columbia | 446 | 11.3 | 0.00 (0.00, 0.00) | 0.00 (0.00, 0.01) | 0.01 (0.00, 0.01) | 0.01 (0.00, 0.01) |
| Cowlitz | 15,131 | 14.4 | 0.03 (0.00, 0.05) | 0.11 (0.00, 0.21) | 0.18 (0.00, 0.36) | 0.21 (0.00, 0.41) |
| Douglas | 4,849 | 11.6 | 0.01 (0.00, 0.02) | 0.03 (0.00, 0.06) | 0.05 (0.00, 0.1) | 0.06 (0.00, 0.12) |
| Ferry | 1,292 | 17.3 | 0.00 (0.00, 0.01) | 0.01 (0.00, 0.02) | 0.02 (0.00, 0.04) | 0.02 (0.00, 0.04) |
| Franklin | 13,558 | 15.2 | 0.01 (0.00, 0.02) | 0.04 (0.00, 0.07) | 0.06 (0.00, 0.13) | 0.07 (0.00, 0.14) |
| Garfield | 227 | 10.3 | 0.00 (0.00, 0.00) | 0.00 (0.00, 0.00) | 0.00 (0.00, 0.00) | 0.00 (0.00, 0.01) |
| Grant | 14,023 | 14.8 | 0.02 (0.00, 0.03) | 0.06 (0.00, 0.13) | 0.11 (0.00, 0.22) | 0.13 (0.00, 0.25) |
| Grays Harbor | 10,037 | 14.4 | 0.02 (0.00, 0.03) | 0.06 (0.00, 0.12) | 0.11 (0.00, 0.21) | 0.12 (0.00, 0.24) |
| Island | 6,291 | 7.8 | 0.00 (0.00, 0.01) | 0.01 (0.00, 0.02) | 0.02 (0.00, 0.04) | 0.02 (0.00, 0.05) |
| Jefferson | 3,945 | 13 | 0.01 (0.00, 0.01) | 0.02 (0.00, 0.05) | 0.04 (0.00, 0.08) | 0.05 (0.00, 0.09) |
| King | 193,603 | 8.9 | 0.17 (0.00, 0.35) | 0.69 (0.00, 1.37) | 1.19 (0.00, 2.36) | 1.36 (0.00, 2.68) |
| Kitsap | 22,404 | 8.7 | 0.03 (0.00, 0.05) | 0.11 (0.00, 0.21) | 0.19 (0.00, 0.37) | 0.21 (0.00, 0.42) |
| Kittitas | 7,808 | 18 | 0.02 (0.00, 0.03) | 0.07 (0.00, 0.13) | 0.11 (0.00, 0.22) | 0.13 (0.00, 0.24) |
| Klickitat | 3,371 | 15.6 | 0.01 (0.00, 0.03) | 0.05 (0.00, 0.10) | 0.09 (0.00, 0.17) | 0.10 (0.00, 0.19) |
| Lewis | 10,069 | 13.1 | 0.01 (0.00, 0.03) | 0.06 (0.00, 0.11) | 0.10 (0.00, 0.20) | 0.11 (0.00, 0.22) |
| Lincoln | 1,307 | 12.5 | 0.00 (0.00, 0.00) | 0.01 (0.00, 0.02) | 0.02 (0.00, 0.03) | 0.02 (0.00, 0.04) |
| Mason | 8,731 | 14 | 0.02 (0.00, 0.04) | 0.07 (0.00, 0.15) | 0.13 (0.00, 0.25) | 0.15 (0.00, 0.28) |
| Okanogan | 8,042 | 19.5 | 0.02 (0.00, 0.03) | 0.06 (0.00, 0.13) | 0.11 (0.00, 0.22) | 0.12 (0.00, 0.24) |
| Pacific | 3,421 | 16 | 0.01 (0.00, 0.01) | 0.03 (0.00, 0.05) | 0.05 (0.00, 0.09) | 0.05 (0.00, 0.11) |
| Pend Oreille | 1,673 | 12.7 | 0.00 (0.00, 0.01) | 0.02 (0.00, 0.03) | 0.03 (0.00, 0.05) | 0.03 (0.00, 0.06) |
| Pierce | 89,714 | 10.4 | 0.10 (0.00, 0.21) | 0.41 (0.00, 0.81) | 0.70 (0.00, 1.39) | 0.80 (0.00, 1.58) |
| San Juan | 1,691 | 10.2 | 0.00 (0.00, 0.00) | 0.01 (0.00, 0.01) | 0.01 (0.00, 0.02) | 0.01 (0.00, 0.03) |
| Skagit | 14,815 | 12 | 0.01 (0.00, 0.02) | 0.05 (0.00, 0.10) | 0.08 (0.00, 0.17) | 0.10 (0.00, 0.19) |
| Skamania | 1,492 | 12.8 | 0.00 (0.00, 0.01) | 0.01 (0.00, 0.03) | 0.02 (0.00, 0.04) | 0.02 (0.00, 0.05) |
| Snohomish | 59,039 | 7.5 | 0.05 (0.00, 0.09) | 0.19 (0.00, 0.37) | 0.32 (0.00, 0.64) | 0.37 (0.00, 0.73) |
| Spokane | 67,236 | 13.7 | 0.13 (0.00, 0.26) | 0.51 (0.00, 1.00) | 0.87 (0.00, 1.68) | 0.99 (0.00, 1.90) |
| Stevens | 6,280 | 14.2 | 0.01 (0.00, 0.03) | 0.05 (0.00, 0.11) | 0.09 (0.00, 0.18) | 0.11 (0.00, 0.20) |
| Thurston | 28,660 | 10.4 | 0.03 (0.00, 0.07) | 0.13 (0.00, 0.26) | 0.23 (0.00, 0.45) | 0.26 (0.00, 0.51) |
| Wahkiakum | 309 | 7.3 | 0.00 (0.00, 0.00) | 0.00 (0.00, 0.00) | 0.00 (0.00, 0.01) | 0.00 (0.00, 0.01) |
| Walla Walla | 7,329 | 13.3 | 0.02 (0.00, 0.03) | 0.06 (0.00, 0.12) | 0.10 (0.00, 0.20) | 0.12 (0.00, 0.23) |
| Whatcom | 30,432 | 14.1 | 0.04 (0.00, 0.09) | 0.17 (0.00, 0.34) | 0.30 (0.00, 0.58) | 0.34 (0.00, 0.66) |
| Whitman | 11,219 | 26.5 | 0.01 (0.00, 0.01) | 0.02 (0.00, 0.05) | 0.04 (0.00, 0.08) | 0.05 (0.00, 0.10) |
| Yakima | 42,755 | 17.4 | 0.08 (0.00, 0.16) | 0.32 (0.00, 0.62) | 0.54 (0.00, 1.06) | 0.62 (0.00, 1.20) |

**Table S5.** Summary of PM_2.5_ concentrations in Washington (correcting monitoring data from Nephelometers) before and during the wildfire episode, 2020.

|  | PM_2.5_ concentrations (𝜇g/m^3^) | | | | | |
| --- | --- | --- | --- | --- | --- | --- |
|  | Mean | SD | Median | Range | Minimum | Maximum |
| Baseline (2019) | 3.1 | 0.8 | 3.0 | 3.6 | 1.4 | 5.0 |
| during wildfire | 83.2 | 34.3 | 73.2 | 183.8 | 25.0 | 208.8 |
| increases | 79.6 | 33.6 | 70.2 | 181.9 | 23.6 | 205.5 |


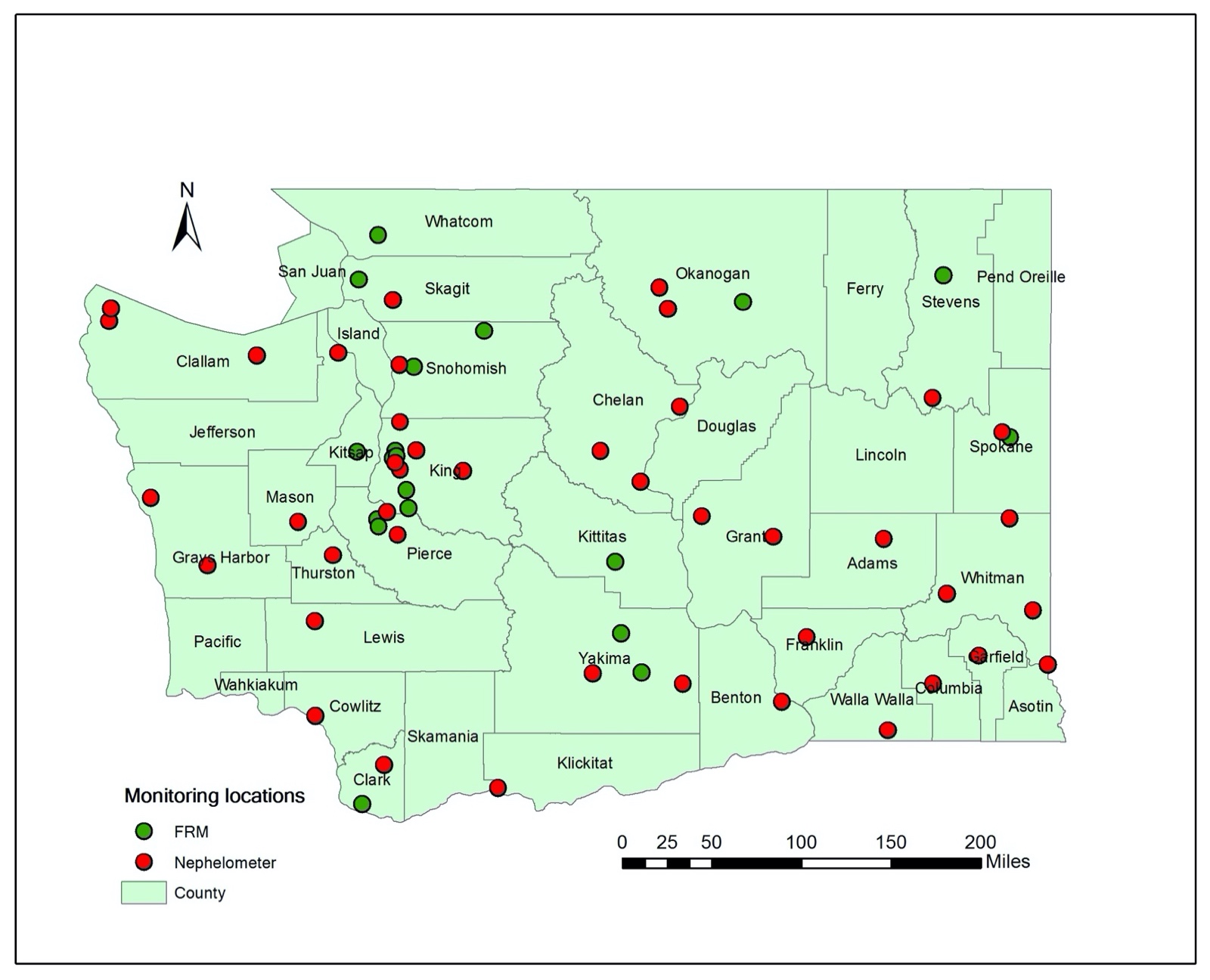


**Figure S1.** The map of the air quality monitoring network in Washington (mobile monitoring available in Ferry, Island, and Pacific County).


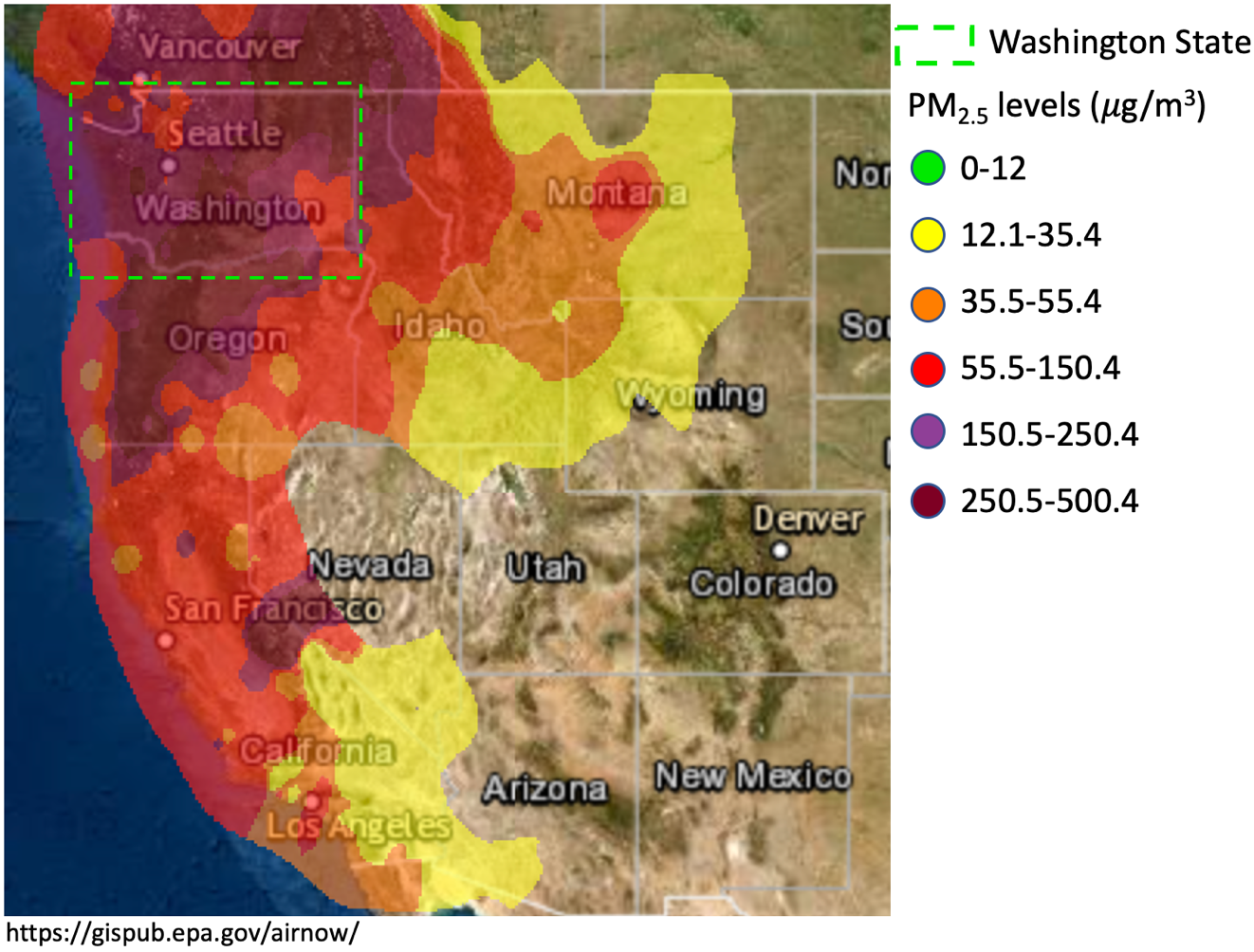


**Figure S2.** The widespread smoke plume across the Washington state on a typical day (September 14, 2020) during the wildfire smoke episode.
